# Tissue-Specific Profiling of *O*-GlcNAcylated Proteins in *Drosophila* Using TurboID-*Cp*OGA^M^

**DOI:** 10.21769/BioProtoc.5234

**Published:** 2025-03-05

**Authors:** Qin Lei, Haibin Yu, Fang Chen, Kai Yuan

**Affiliations:** 1 Hunan Key Laboratory of Molecular Precision Medicine, Department of Oncology, Xiangya Hospital, Central South University, Changsha, China; 2 Center for Medical Genetics, School of Life Sciences, Central South University, Changsha 410008, China; 3 Furong Laboratory, Changsha, China; 4 National Clinical Research Center for Geriatric Disorders, Xiangya Hospital, Central South University, Changsha, China; 5 The Biobank of Xiangya Hospital, Central South University, Changsha, China

**Keywords:** *Drosophila*, Protein *O-*GlcNAcylation, TurboID proximity labeling, Tissue-specific analysis

## Abstract

Protein *O-*GlcNAcylation is a prevalent and dynamic post-translational modification that targets a multitude of nuclear and cytoplasmic proteins. Through the modification of diverse substrates, *O-*GlcNAcylation plays a pivotal role in essential cellular processes, including transcription, translation, and protein homeostasis. Dysregulation of *O-*GlcNAc homeostasis has been implicated in a variety of diseases, including cardiovascular diseases, cancer, and neurodegenerative diseases. Studying *O-*GlcNAcylated proteins in different tissues is crucial to understanding the pathogenesis of these diseases. However, identifying phenotype-relevant candidate substrates in a tissue-specific manner remains unfeasible. We developed a novel tool for the analysis of *O-*GlcNAcylated proteins, combining a catalytically inactive *Cp*OGA mutant *Cp*OGA^CD^ and TurboID proximity labeling technology. This tool converts *O-*GlcNAc modifications into biotin labeling, enabling the enrichment and mass spectrometry (MS) identification of *O-*GlcNAcylated proteins in specific tissues. Meanwhile, TurboID-*Cp*OGA^DM^, which carries two point mutations that inactivate both its catalytic and binding activities toward *O*-GlcNAc modification, was used as a control to differentiate *O-*GlcNAc-independent protein–protein interactions. We have successfully used TurboID-*Cp*OGA^CD/DM^ (TurboID-*Cp*OGA^M^) to enrich *O*-GlcNAc proteins in *Drosophila* combining the UAS/Gal4 system. Our protocol provides a comprehensive workflow for tissue-specific enrichment of candidate *O-*GlcNAcylated substrates and offers a valuable tool for dissecting tissue-specific *O*-GlcNAcylation functions in *Drosophila*.

Key features

• Innovative approach to studying *O-*GlcNAcylation: Combines a catalytically inactive *Cp*OGA mutant (*Cp*OGA^CD^), TurboID proximity labeling technology, and the UAS/Gal4 system for tissue-specific analysis.

• Tissue-specific focus: Enables enrichment and mass spectrometry (MS) identification of *O-*GlcNAcylated proteins in specific tissues of *Drosophila*.

• Biotin labeling conversion: Converts *O-*GlcNAc modifications into biotin tags, facilitating downstream enrichment and analysis.

• Powerful tool for understanding the role of *O-*GlcNAcylation in cellular processes and its involvement in diseases such as cardiovascular diseases, cancer, and neurodegenerative disorders.

## Graphical overview


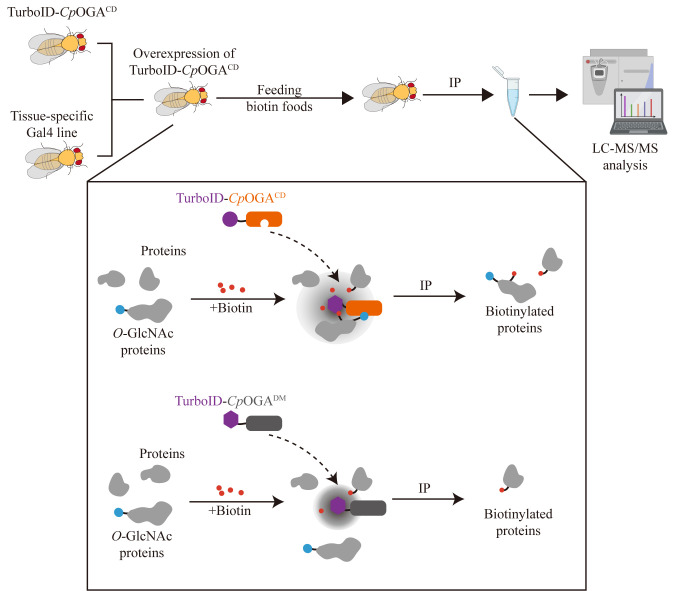


**Schematic of the TurboID-*Cp*OGA^M^ approach.** Cross TurboID-*Cp*OGA^M^ with different Gal4 driver lines to achieve tissue-specific overexpression of TurboID-*Cp*OGA^M^ in offspring. Upon biotin addition, TurboID catalyzes the biotinylation of *O*-GlcNAcylated proteins bound to *Cp*OGA^CD^, enabling their enrichment through streptavidin pull-down for subsequent mass spectrometry (MS) analysis. TurboID-*Cp*OGA^DM^, which lacks both catalytic and *O*-GlcNAc recognition, is used as a negative control to distinguish *O-*GlcNAc-independent protein–protein interactions. Red represents biotin; blue represents *O*-GlcNAc proteome; gray represents proteins.

## Background

*O-*GlcNAcylation is a post-translational modification where a single N-acetylglucosamine (GlcNAc) molecule is attached to the hydroxyl oxygen atom of serine/threonine residues on proteins [1]. It is regulated by two evolutionarily conserved enzymes: *O-*GlcNAc transferase (OGT), which adds the *O-*GlcNAc modification, and *O-*GlcNAcase (OGA), which removes it [2]. *O*-GlcNAcylation plays a pivotal role in the regulation of fundamental cellular processes, encompassing transcription, translation, and protein homeostasis [3]. Identifying *O*-GlcNAcylated substrates in different tissues is critical for understanding disease mechanisms.

Profiling *O*-GlcNAc-modified proteins via mass spectrometry (MS) requires enrichment due to their low abundance and diversity. Enrichment strategies fall into two categories. One involves the direct capture of *O*-GlcNAcylated proteins using antibodies or lectins that specifically recognize the GlcNAc moiety. Various general antibodies have been developed targeting different *O-*GlcNAcylated proteins and peptide sequences, such as RL2 [4], which was generated from a glycosylated peptide sequence of nucleoporins, and CTD110.6 [5], derived from a glycosylated peptide sequence in the C-terminal domain of RNA polymerase II. Wheat germ agglutinin (WGA), a lectin recognizing *O-*GlcNAc and other polysaccharides, has weaker interactions [6]. The second enrichment strategy employs chemoenzymatic or metabolic labeling [7]. This involves introducing azido-modified sugar analogs, such as N-azidoacetylglucosamine (GlcNAz) and N-azidoacetylgalactosamine (GalNAz), to tag proteins with biotin through click chemistry. It presents many advantages such as low background, correlation of cell survival, and simplicity of steps for cell labeling [8,9], though it may lead to non-specific labeling of other glycosylation processes.

It is worth noting that different *O*-GlcNAc profiling strategies yield distinct sets of *O*-GlcNAcylated proteins, likely due to the dynamic nature of *O*-GlcNAc cycling and potential biases in substrate selection inherent to each method. Despite these differences, these advancements have significantly expanded the known *O*-GlcNAcome over the last 30 years. However, none of these techniques have been employed for the tissue-specific identification of *O*-GlcNAcylated proteins.

*Cp*OGA, a homolog of OGA from *Clostridium perfringens*, has been deeply studied to recognize *O-*GlcNAcylated proteins. *Cp*OGA^M^ refers to the two fusion proteins, *Cp*OGA^CD^ and *Cp*OGA^DM^. The *Cp*OGA^CD^ variant has the catalytic residue at position 298 (aspartic acid, Asp) mutated to asparagine (Asn), abolishing its catalytic activity but retaining nanomolar affinity for *O-*GlcNAc-modified peptides or proteins [10]. A further mutation at position 401 (Asp → Ala) creates the double mutant *Cp*OGA^DM^, which lacks both catalytic activity and *O*-GlcNAc recognition [10]. Thus, the *Cp*OGA^CD^ can specifically recognize *O-*GlcNAcylated proteins in cells. However, it cannot achieve the goal of enriching tissue-specific proteins. To enable the study of tissue-specific *O-*GlcNAcylated proteins, we developed a novel tool that combines the highly efficient biotin ligase TurboID with *Cp*OGA^CD^ according to the principle of proximity labeling, resulting in the construct TurboID-*Cp*OGA^M^. Thus, TurboID-*Cp*OGA^M^ can efficiently bind to *O-*GlcNAcylated proteins specifically in cells. This approach deeply facilitates the selective enrichment of *O-*GlcNAcylated proteins. We also applied the GAL4/UAS system, a powerful tool for targeted gene expression [11] in a *Drosophila* model, to identify tissue-specific *O-*GlcNAcylated proteins.

This protocol outlines the construction of the TurboID-*Cp*OGA^M^ expression system, isolation and validation of biotinylated proteins, preparation of samples for mass spectrometry, and data analysis.

## Materials and reagents

**Fly stocks**

1. *Da-Gal4* (BDSC, #95282)

2. *Elav-Gal4* (BDSC, #8765)

3. *OK107-Gal4* (BDSC, #854)

4. *C232-Gal4* (BDSC, #30828)

5. *GMR14H04-Gal4* (BDSC, #48655)

6. *GMR33H10-Gal4* (BDSC, #49762)

7. *201Y-Gal4* (BDSC, #4440)

8. *UAS-HA-TurboID-CpOGA^CD^* (generated in this study)

9. *UAS-HA-TurboID-CpOGA^DM^* (generated in this study)

10. *w1118* (gift from Kun Xia’s lab)

11. *Tub-Gal80^ts^* (gift from Jun Ma’s lab)

12. *sco/cyo; TM3/TM6B* (gift from Kun Xia’s lab)

**Reagents**

1. Corn flour (Tengzhou Zhengda Grain Processing Factory)

2. Agar (Solarrbio, catalog number: A8190)

3. Glucose (Sinopharm, catalog number: 14431-43-7)

4. Sucrose (Sinopharm, catalog number: 10021418-500 g)

5. Yeast powder (AB Mauri Yeast Co., Ltd)

6. Agarose (Qingke Bio, catalog number: TSJ001)

7. Tris (Coolaer, catalog number: CT11411)

8. Yeast extract (OXIOD, catalog number: LP0021-500 g)

9. Reverse Transcriptase cDNA Kit (Thermo Fisher Scientific, catalog number: K16225)

10. Plasmid Extraction kit (Magen, catalog number: p1001-03c)

11. Gel Extraction kit (Magen, catalog number: D2111-02-100)

12. PCR Purification kit (Thermo, Invitrogen, catalog number: K310001)

13. BCA Protein Assay kit (Beyotime, catalog number: p0009)

14. Protease inhibitor (cocktail) (Sigma, catalog number: P8340-5 mL)

15. 4% PFA (Servicebio, catalog number: G1101)

16. KCl (GuoYao, catalog number: 10016318-500 g)

17. Na_2_CO_3 _(Energy Chemical, catalog number: E0103975000)

18. Triton X-100 (Sigma, catalog number: T8200)

19. DAPI (Sigma, catalog number: D9542)

20. Streptavidin magnetic beads (MCE, catalog number: HY-K0208)

21. Biotin (Sigma, catalog number: B4501-100MG)

22. BSA (Biofroxx, catalog number: 4240GR005)

23. Coomassie Brilliant Blue (Solarbio, catalog number: C8430-10G)

24. IAA (Merck, catalog number: I6125)

25. DTT (Solarbio, catalog number: D8220-5 g)

26. Trypsin (BI, catalog number: 03-050-1ACS)

27. Acetonitrile (Energy Chemical, catalog number: A011968)

28. Sealing reagent (Solarbio, catalog number: S2100)

29. Stage tip (Thermo Fisher Scientific, catalog number: 87782)

30. DMSO (Sigma, catalog number: D2650-100 mL)

31. Urea powder (Solarbio, catalog number: U8020-1 kg)

32. Formic acid (Sigma, catalog number: 64-18-6)

33. EDTA (Sigma, catalog number: V900106)

34. Bromophenol Blue (Guoyao, catalog number: 71008060)

**Solutions**

1. 10× PBS (see Recipes)

2. 0.3% PBTA (see Recipes)

3. 0.1% PBST (see Recipes)

4. 0.3% PBST (see Recipes)

5. 10% SDS (see Recipes)

6. 5% skim milk (see Recipes)

7. RIPA buffer (see Recipes)

8. Primary antibody solution (see Recipes)

9. Fly food (see Recipes)

10. Fly food containing biotin (see Recipes)

11. Secondary antibody solution (see Recipes)

12. 10 mM DTT (see Recipes)

13. 1 M KCl (see Recipes)

14. 0.1 M Na_2_CO_3_ (see Recipes)

15. 2 M urea (see Recipes)

16. 10 mM Tris-HCl (see Recipes)

17. 80% acetonitrile (see Recipes)

18. 0.1% formic acid (see Recipes)

19. Protein loading buffer (see Recipes)

**Recipes**

**1. 10× PBS**

Weigh 2.0 g of KCl, 80.0 g of NaCl, 2.4 g of KH_2_PO_4_, and 14.4 g of Na_2_HPO_4_. Dissolve these salts in ddH_2_O to a final volume of 1 L. Mix thoroughly and sterilize the solution under high pressure. Store at room temperature after sterilization.

**2. 0.3% PBTA**

Weigh 0.06 g of BSA and dissolve it in 0.3% PBST to a final volume of 20 mL.

**3. 0.1% PBST**

Add 250 μL of 20% Triton X-100 and 5 mL of 10× PBS to ddH_2_O to a final volume of 50 mL. Filter the solution using a sterile filter to remove any bacteria and store it at room temperature.

**4. 0.3% PBST**

Add 750 μL of 20% Triton X-100 and 5 mL of 10× PBS to ddH_2_O to a final volume of 50 mL. Filter the solution using a sterile filter to remove any bacteria and store it at room temperature.

**5. 10% SDS**

Dissolve 100.0 g of SDS in 400 mL of ddH_2_O and adjust the volume to 500 mL. Store the solution at room temperature or at 4 °C.

**6. 5% skim milk**

Dissolve 2.5 g of skim milk powder completely in 30 mL of 0.1% PBST and add 0.1% PBST to a final volume of 50 mL.

**7. RIPA buffer**

Weigh 3.03 g of Tris-HCl, 4.35 g of NaCl, 5 mL of NP40/Triton X-100, 2.5 g of sodium deoxycholate, and 0.5 g of SDS. Add ddH_2_O to a final volume of 500 mL and store the solution protected from light at -20 °C.

**8. Primary antibody solution**

Weigh 0.25 g of BSA and dissolve it in 0.1% PBST to a final volume of 5 mL. Then, add 5 µL of the primary antibody at a 1:1,000 dilution ratio.

**9. Fly food**

First, dissolve 16.8 g of agar in 500 mL of distilled water by heating and stirring until fully dissolved. Next, gradually add 116.4 g of corn flour, 47.4 g of sucrose, 94.8 g of glucose, and 45 g of yeast, stirring continuously to prevent clumping. Adjust the total volume to approximately 2,000 mL by adding distilled water. Allow the mixture to cool to below 60 °C, then add 22.5 mL of 10% methylparaben (dissolved in ethanol) as a preservative. Stir thoroughly and dispense the mixture into vials or bottles. Let it solidify at room temperature before storing it at 4 °C or using it for fly cultures.

**10. Fly food containing biotin**

Dissolve biotin powder (approximately 48.4 mg) in approximately 1 mL of preheated DMSO at 80 °C to prepare a biotin stock solution (200 mM). This solution should be stored at -20 °C for no more than 3 months. Heat the fly food in a microwave until it is completely dissolved, then add the biotin stock solution to achieve a final biotin concentration of 200 μM in the fly food.

**11. Secondary antibody solution**

Add 0.5 µL of the secondary antibody at a 1:10,000 dilution ratio in 5% skim milk.

**12. 10 mM DTT**

Weigh 46.275 mg of DTT powder and dissolve it in ddH_2_O to a final volume of 30 mL.

**13. 1 M KCl**

Weigh 2.2365 g of KCl and dissolve it in ddH_2_O to a final volume of 30 mL.

**14. 0.1 M Na_2_CO_3_**

Weigh 317.97 mg of Na_2_CO_3_ and dissolve it in ddH_2_O to a final volume of 30 mL.

**15. 2 M urea**

Weigh 3.6 g of urea powder and dissolve it in ddH_2_O to a final volume of 30 mL.

**16. 10 mM Tris-HCl**

Add 1 mL of 1 M Tris-HCl solution into a 100 mL volumetric flask. Dilute to the mark with ddH_2_O to make up to 100 mL.

**17. 80% acetonitrile**

Transfer 80 mL of acetonitrile and 100 μL of formic acid into a 100 mL volumetric flask, then dilute to the mark with ddH_2_O to reach a final volume of 100 mL. Ensure that this procedure is conducted in a fume hood.

**18. 0.1% formic acid**

Accurately pipette 0.1 mL of formic acid and transfer it into a volumetric flask. Add ddH_2_O to a final volume of 100 mL.

**19. Protein loading buffer**

Weigh 24.024 g of urea, 50 g of sucrose, 0.0372 g of EDTA, and 100 μL of Bromophenol Blue in a 100 mL volumetric flask, then dilute to the mark with ddH_2_O to a final volume of 100 mL.

**Antibodies**

1. Streptavidin-HRP (GenScript, catalog number: M00091)

2. Streptavidin-Cy3 (BioLegend, catalog number: 405215)

3. Anti-*O*-Linked N-Acetylglucosamine antibody (RL2) (mouse) (Abcam, catalog number: ab2739)

4. Anti-HA Antibody Rabbit mAb (C29F4) (Cell Signaling Technology, catalog number: 3724)

5. α-Tubulin (DM1A) Mouse mAb (Cell Signaling Technology, catalog number: 12351S)

6. Anti-Mouse or Rabbit secondary antibodies (Thermo Fisher Scientific, catalog number: 31460-2 mL)

7. Goat Anti-Mouse lgG (H+L) secondary antibody (Thermo Fisher Scientific, catalog number: A11001)

**Materials**

1. 1.5 mL EP tubes (Axygen, catalog number: MCT-150-C)

2. PCR tubes (Axygen, catalog number: PCR-02-c)

3. Tips (Pullen, catalog number: PA02002)

4. Cationic slides (Shitai, catalog number: 3102100AO)

5. Round coverslips (Fisherbrand, catalog number: 10212020C)

6. Precision tweezers (RWD, catalog number: F11029-11)

7. Lint-free paper (KIMWIPES, catalog number: 34155)

8. Silanized coverslips (SHITAI, catalog number: 80340-3110)

9. Pipettes (Beaver, catalog number: 43003)

10. Liquid nitrogen storage boxes (NALGENE, catalog number: 5026-0909)

11. PVDF (Millipore, catalog number: IPVH00010)

12. CO_2_ pad (Zhuo Kai, catalog number: 12.6*8.2*1.3 cm)

## Equipment

1. *Drosophila* incubator (TST, catalog number: GH4500)

2. Agarose Gel Electrophoresis kit (Tanon, catalog number: VE1)

3. Protein electrophoresis apparatus (Tanon, catalog number: VE180)

4. Transfer apparatus (Tanon, catalog number: VE186)

5. PCR machine (Bio-Rad, catalog number: QUANTSTUDIO 3)

6. Stereomicroscope (Motic, catalog number: MZ61)

7. Confocal microscope (Zeiss, catalog number: LSM880)

8. Fluorescence stereomicroscope (Leica, catalog number: M205FCA)

9. CO_2_ cell culture incubator (Thermo Fisher Scientific, catalog number: 3111)

10. Q Exactive HF-X mass spectrometer (Thermo Fisher Scientific, catalog number: 0726042 10)

11. Easy nLC 1200 system (Thermo Fisher Scientific, catalog number: LC1400)

## Software and datasets

1. MaxQuant

2. Perseus

3. Adobe Illustrator

4. GraphPad Prism

5. ZEISS Zen

## Procedure

**A. Construction of the TurboID-*Cp*OGA^M^ expression system**

**Figure 1.**
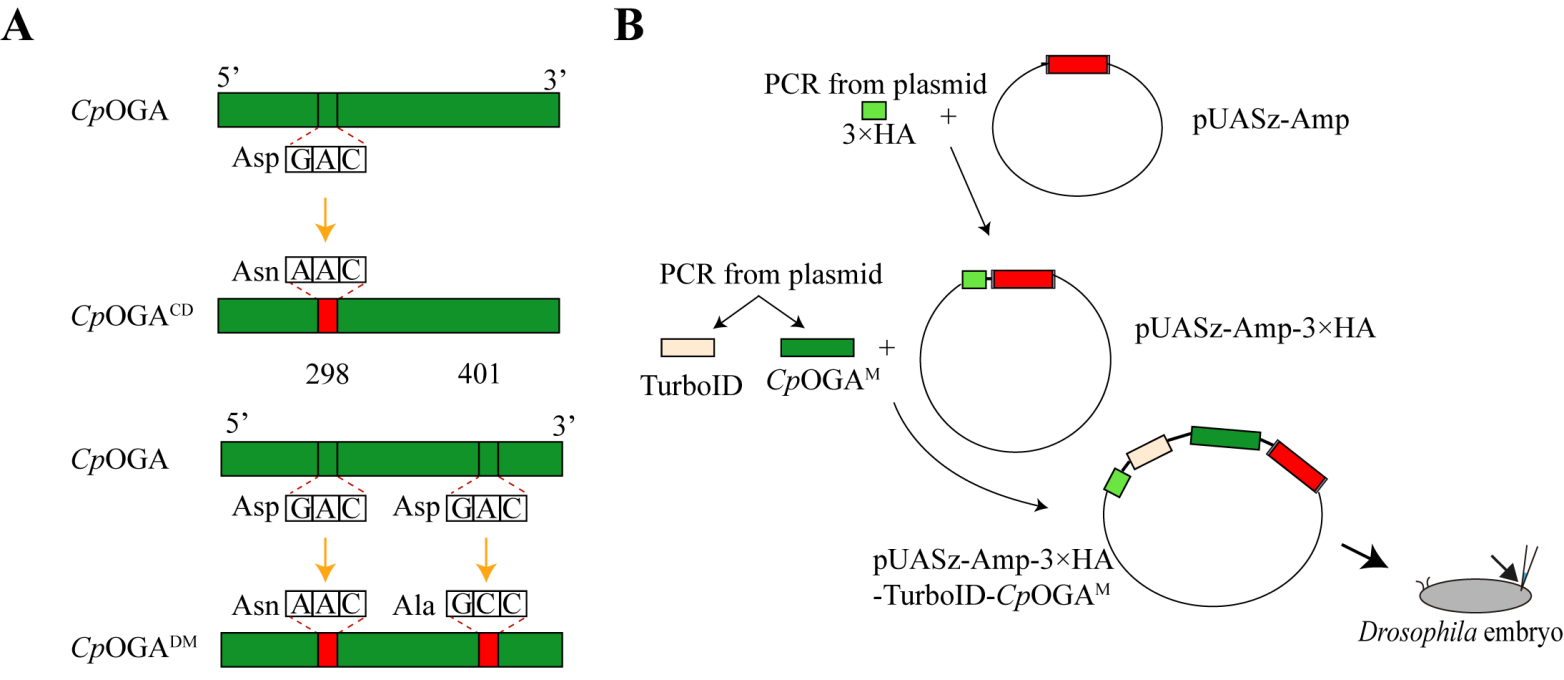
Graphic map of *Cp*OGA^M^ and the whole plasmid. (A) In *Cp*OGA^CD^, the catalytic residue at position 298 (aspartic acid, Asp) is mutated to asparagine (Asn). In *Cp*OGA^DM^, the catalytic residue at position 298 (Asp) is mutated to Asn, and the residue at position 401 (Asp) is mutated to alanine (Ala). (B) Schematic diagram of complete plasmid construction.

1. Construction of UAS-TurboID-*Cp*OGA^M^ plasmids

a. Synthesize the *Cp*OGA^CD^ sequence [12] and optimize its codons for *Homo sapiens* and *Drosophila* using *Jcat* [13].

b. Generate *Cp*OGA^DM^ sequences via point mutation based on *Cp*OGA^CD^ and synthesize the TurboID sequences [14].

c. TurboID fragment and *Cp*OGA^M^ fragment were cloned into the pUASz-HA vector using Gibson Assembly (Figure 1).

**Figure 2.**
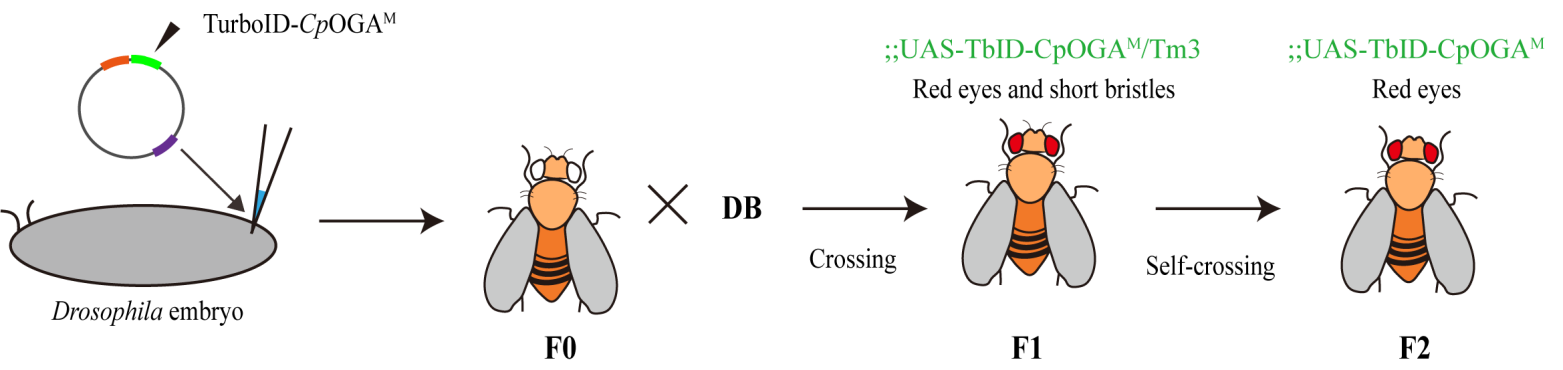
Diagram of the conditional TurboID-*Cp*OGA^M^ overexpression construct in *Drosophila.* TurboID-*Cp*OGA^M ^plasmids were purified and injected into attP2 embryos. F0 males were crossed with balancer females. Red-eyed, short-bristled F1 progeny were selected and crossed with balancer flies, and stable transgenic lines were established.

2. Transgenic fly generation (Figure 2)

a. Purify TurboID-*Cp*OGA^M^ plasmids to remove endotoxin and improve the survival rate of embryos after injection. Then, inject them into embryos that have been collected in advance at the pre-germ-layer stage of the attP2 line following standard injection procedures (Zhuhai United Huayi Company). The *Drosophila* embryo injection process involves embryo synchronization, collection of embryos, pulling of injection capillaries, preparation of the injection mixture, and injection using the Eppendorf Femtojet. After injection, the embryos are transferred, and the emerged F0 flies are collected and used for setting up crosses to ensure gene expression and phenotypic analysis.

b. Incubate injected embryos at 25 °C to allow development.

c. After emergence, cross F0 male flies with balancer female flies in a 3:1 or 4:1 ratio.

d. Approximately 10 days later, separate male and female transgenic offspring, selecting virgin females and males displaying red eyes and short bristles.

e. Cross the selected F1 progeny with balancer flies to establish a stable transgenic line.

f. Identify transgenic offspring through genomic PCR and sequencing to confirm integration.

g. Select virgin females and males from the F2 progeny that lack short bristles as the desired homozygous flies with conditional overexpression of TurboID-*Cp*OGA^M^.

**Figure 3.**
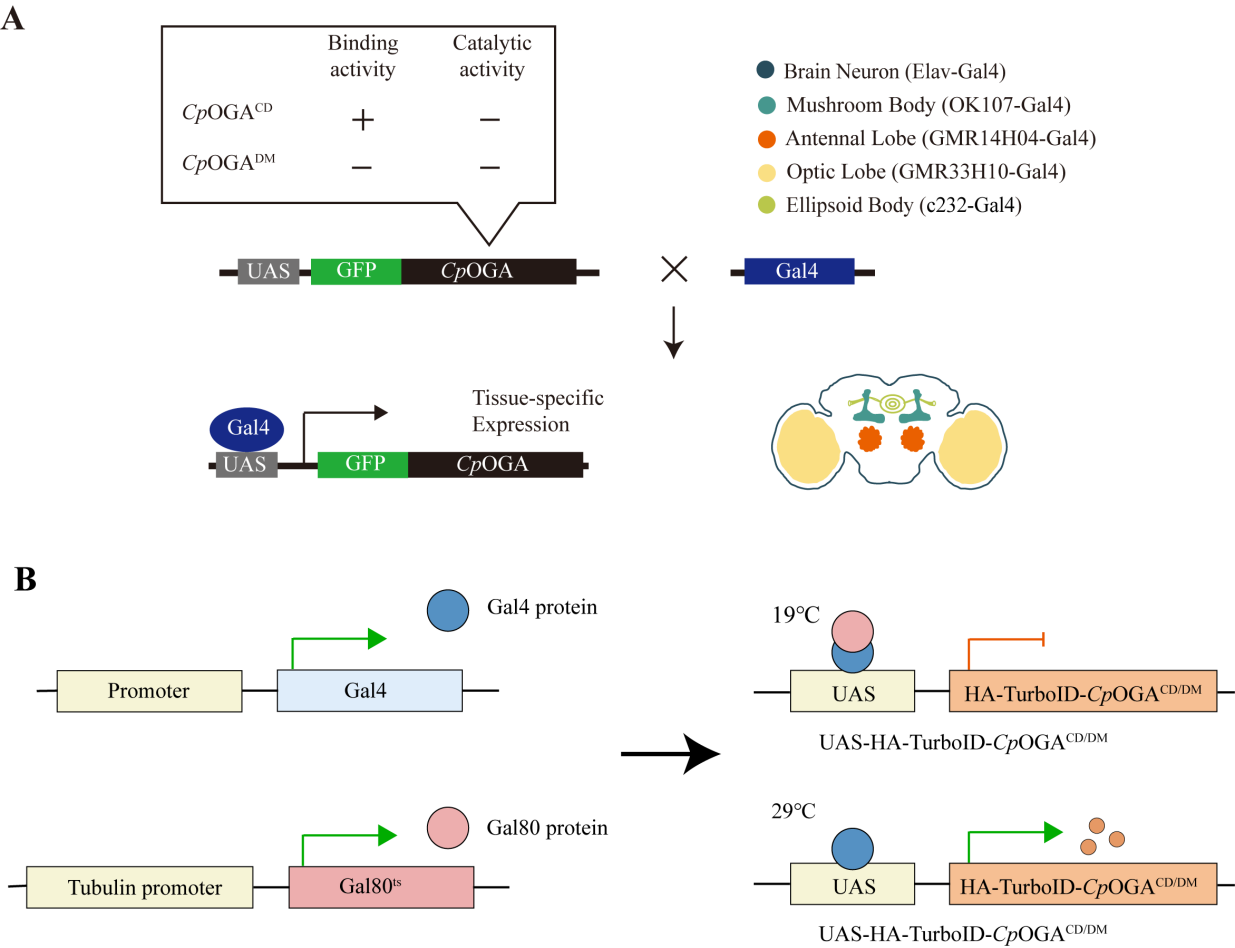
Scheme for expression of *Cp*OGA^M^ in various tissues using different Gal4 drivers. (A) Expression of GFP and *Cp*OGA (*Cp*OGA^WT^ exhibits both binding and catalytic activities, whereas *Cp*OGA^DM^ lacks both) in specific regions of the *Drosophila* brain using the Gal4/UAS system. *Cp*OGA can be expressed in brain neurons (Elav-Gal4), mushroom body (OK107-Gal4), antennal lobe (GMR14H04-Gal4), optic lobe (GMR33H10-Gal4), and ellipsoid body (c232-Gal4). This figure is reprinted from Figure 1A in our previous publication [15]. (B) Schematic model of Gal4/Gal80^ts^ system.

3. Construction of tissue-specific TurboID-*Cp*OGA^M^ system (Figure 3A)

a. Prepare biotin-containing fly food (see Recipes).

b. Collect virgin female flies with the genotype (UAS-TurboID-*Cp*OGA^M^ and Gal4 line) three times daily from the pupal stage. Cross virgin females with males of the same genotype at a 3:1 ratio. Maintain the crosses at 25 °C for 5 days. Transfer the crossed *Drosophila* into new tubes, leaving only the embryos in the original tube. The tube with embryos should be incubated at 25 °C until the next generation emerges as adult flies. Transfer newly emerging adult fruit flies to food supplemented with 100 μM biotin at 25 °C.

c. To precisely control the timing of Gal4 expression in *Drosophila*, we employ the temperature-sensitive Gal80 (Gal80^ts^) to temporally control *Cp*OGA expression, restricting it until adulthood. Gal80^ts^ is a temperature-sensitive variant of the Gal80 protein, which normally represses Gal4 activity in the UAS /Gal4 system. At permissive temperatures (typically, 18–25 °C), Gal80^ts^ inhibits Gal4, preventing the expression of UAS-driven genes. At restrictive temperatures (typically, 29–30 °C), Gal80^ts^ becomes inactivated, allowing Gal4 to activate UAS-targeted gene expression. This system enables conditional, temperature-controlled expression of genes in specific tissues or developmental stages (Figure 3B). For experiments that require the use of Gal80, collect virgin female flies with the genotype (UAS-TurboID-*Cp*OGA^M^; tub-Gal80^ts^ and Gal4 line) three times daily from the pupal stage. Cross virgin females with males of the same genotype at a 3:1 ratio. Maintain the crosses at 19 °C, replacing the culture tubes every three days. After 10–11 days, transfer newly emerged adult flies to food supplemented with 100 μM biotin. Before conducting the experiment, transfer the fruit flies to a 29 °C environment for 48 h in advance to activate the expression of TurboID-*Cp*OGA^M^.

**B. Isolation and validation of biotinylated proteins in *Drosophila***

**Figure 4.**
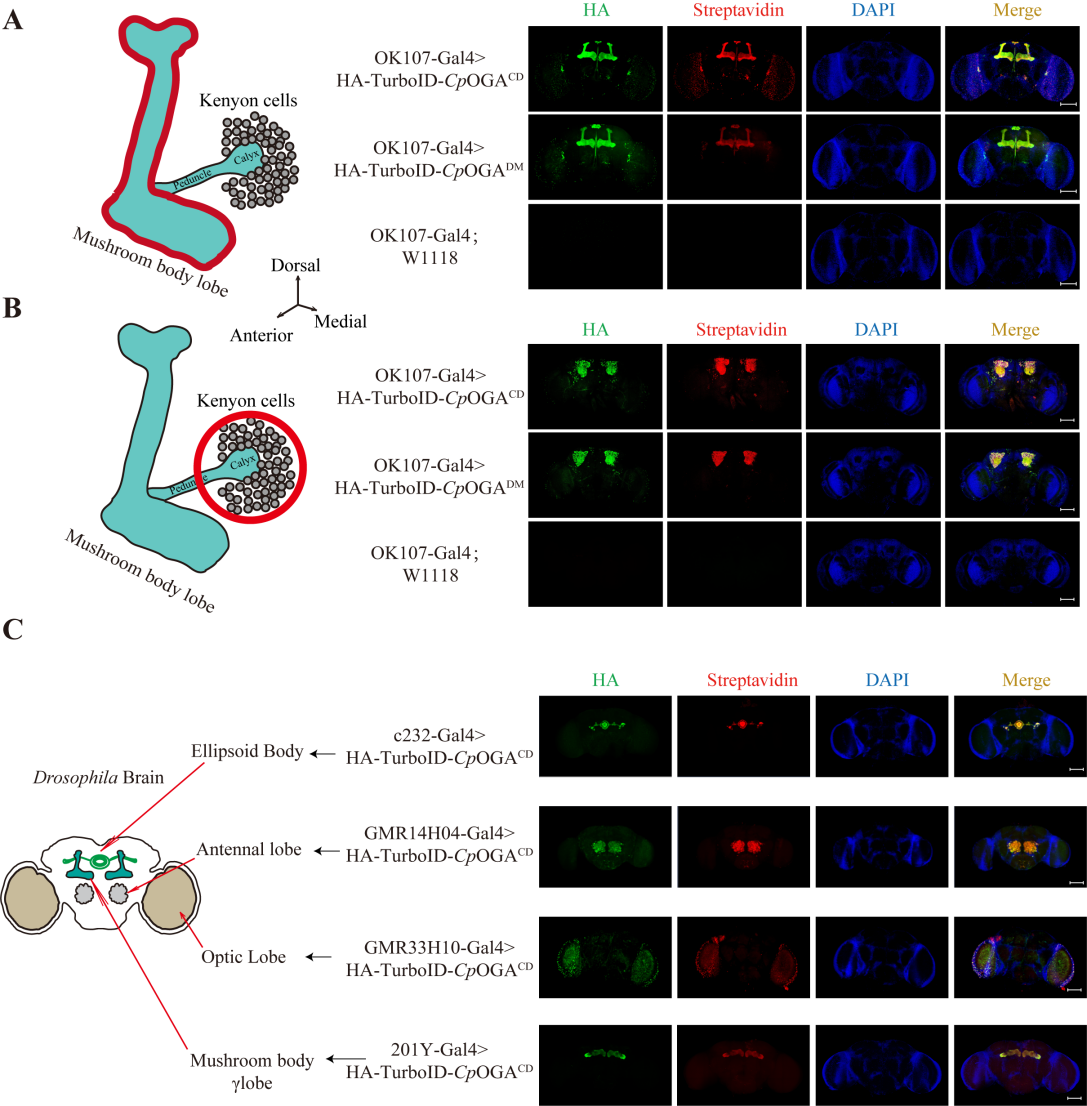
Tissue-specific biotinylation labeling with TurboID-*Cp*OGA^M^. (A–C). Immunofluorescence images showing tissue-specific expression of TurboID-*Cp*OGA^M^ in different regions of the *Drosophila* brain, driven by various Gal4 drivers. From top to bottom: adult mushroom body (OK107-Gal4), ellipsoid body (c232-Gal4), antennal lobe (GMR14H04-Gal4), optic lobe (GMR33H10-Gal4), and mushroom body γ lobe (201Y-Gal4). *w1118* (white eye, X-linked mutation) wild-type *Drosophila* serves as a control. Green indicates HA-tagged TurboID-*Cp*OGA^M^, red represents streptavidin-labeled biotinylated modifications, and blue represents nuclei stained with DAPI. Scale bar = 100 μm.

1. Verification of brain tissue-specific expression of TurboID-*Cp*OGA^M^ by immunofluorescence (Figure 4)

a. To anesthetize adult fruit flies (approximately 10 days old), place them on a CO_2_ pad.

b. On the silicone pad, use tweezers to separate the head of the fruit fly from the thorax and abdomen, and place the head in 4% PFA for pre-fixation for 5 min.

c. Dissect adult brains in PBS and transfer them to a PCR tube containing 200 μL of fresh PBS.

d. Remove the PBS, add 200 μL of 4% PFA to the samples, and fix at room temperature for 1 h.

e. Discard the 4% PFA and wash the samples three times with 0.3% PBST, allowing 10 min per wash.

f. Remove the 0.3% PBST, add 0.3% PBTA, and permeabilize and block the samples at room temperature for 1.5 h.

g. Discard the 0.3% PBTA, add the pre-prepared primary antibody solution, and incubate overnight with gentle rotation at 4 °C.

h. After primary antibody incubation, wash the samples three times with 0.3% PBST, 10 min per wash.

i. Remove the 0.3% PBST, add the prepared secondary antibody solution, and incubate at room temperature with gentle rotation, protected from the light, for 1 h.

j. Discard the secondary antibody solution and add DAPI staining solution to the samples for 15 min.

k. Remove the DAPI staining solution and wash the samples three times with 0.3% PBST, allowing 10 min per wash.

l. After the final wash, discard the 0.3% PBST, add a sealing agent, and, once sealed, observe the samples under a confocal microscope.

**Figure 5.**
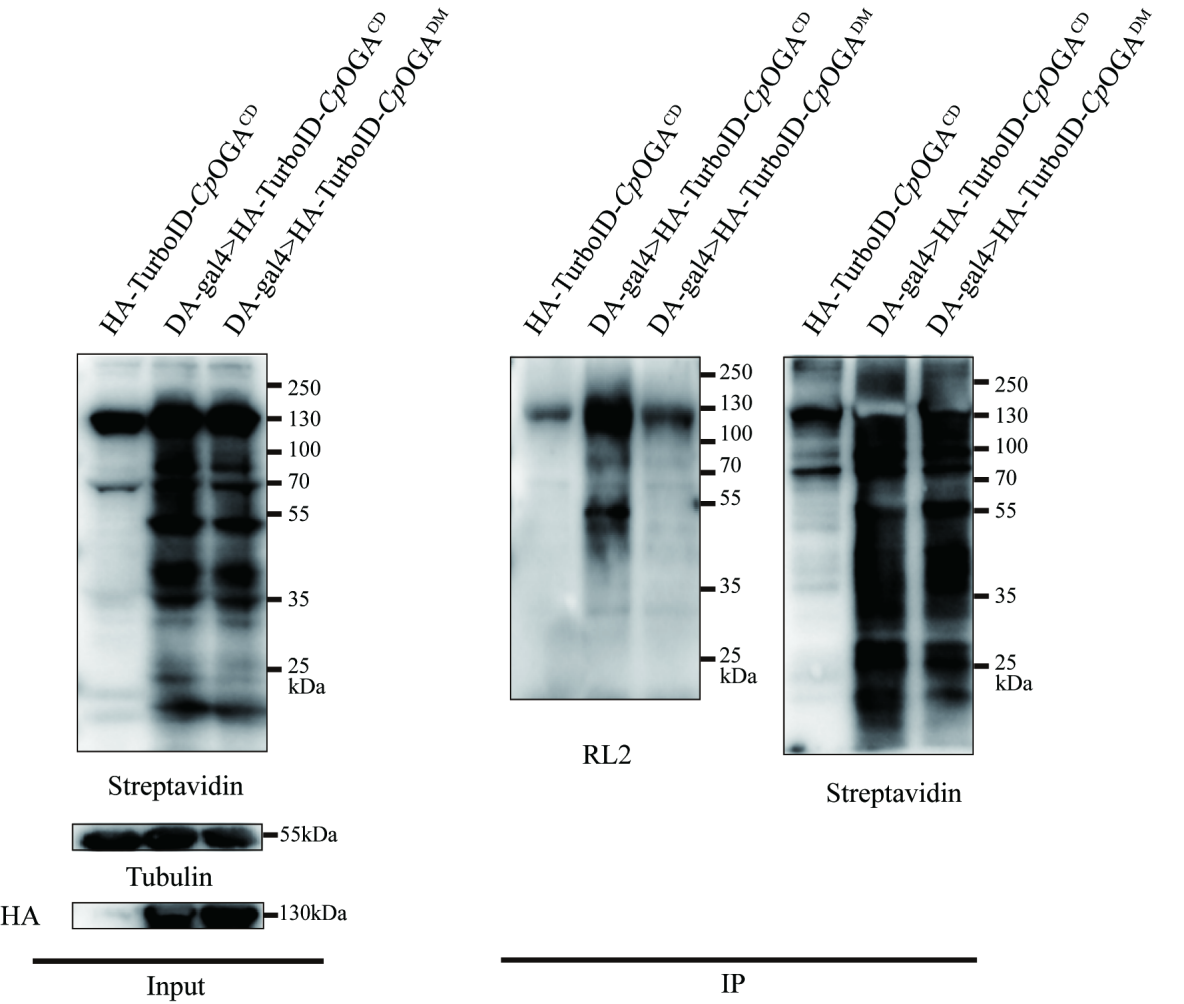
TurboID-*Cp*OGA^M^ can significantly enrich *O*-GlcNAcylated proteins in *Drosophila.* The results of the immunoprecipitation experiment of adult *Drosophila* expressing TurboID-*Cp*OGA^M^ driven by DA-Gal4 were obtained from tissue lysates. Streptavidin was used to label biotinylated proteins, RL2 was used to label *O*-GlcNAcylated proteins, and tubulin was used as the internal reference.

2. Immunoprecipitation with streptavidin magnetic beads (Figure 5) (taking the whole body of fruit flies as an example)

a. Anesthetize 20 adult fruit flies (approximately 10 days old) on a CO_2_ pad. Then, on a silicone pad, use tweezers to separate the heads from the thorax and abdomen. Transfer the heads to a microcentrifuge tube containing 200 μL of ice-cold PBS to preserve tissue integrity.

b. After completing the dissections, add RIPA buffer containing protease inhibitors to the collected samples.

c. Homogenize thoroughly with a pestle, then sonicate each sample three times for 5 s. Incubate on ice for 20 min, vortexing twice during this period.

d. After centrifugation at 13,000× *g* for 30 min at 4 °C, transfer the supernatants to new tubes.

e. Perform a BCA protein assay to determine protein concentration for each sample. Prepare a standard curve using protein concentrations of 0, 0.6, 0.8, 1.0, 1.2, 1.6, and 2.0 μg/μL. Dilute the sample to be tested, incubate with BCA reagents at 37 °C for 20 min, and measure protein concentration.

f. Mix proteins with an equal volume of SDS sample buffer (2% β-Mercaptoethanol) and boil for 10 min at 95 °C.

g. Load and run the samples on a 10% (w/v) SDS gel (90 V, 30 min; 120 V, 1 h).

h. After gel electrophoresis, transfer the samples to a PVDF membrane using standard equipment and protocols.

i. Block the membrane with 5% (w/v) BSA for 1 h, incubate with primary antibodies overnight at 4 °C, and then incubate with secondary antibodies (1:500) for 1 h at room temperature.

j. Detect the signal using ECL substrates. Primary antibodies were dissolved in 5% (w/v) BSA; the following dilutions were made: streptavidin-HRP (1:2,000), RL2 (1:1,000), HA (1:3,000), and Tubulin (1:3,000).

3. Pull-down of biotinylated proteins (taking the whole body of fruit flies as an example)

a. Anesthetize 20 adult fruit flies (approximately 10 days old) on a CO_2_ pad. Then, on a silicone pad, use tweezers to separate the heads from the thorax and abdomen. Transfer the heads to a microcentrifuge tube containing 200 μL of ice-cold PBS to preserve tissue integrity.

b. After completing the dissections, add RIPA buffer containing protease inhibitors to the collected samples. Homogenize thoroughly with a pestle, then sonicate each sample three times for 5 s. Incubate on ice for 20 min, vortexing twice during this period.

c. Clarify lysates by centrifugation at 17,000× *g* for 30 min at 4 °C and determine protein concentration using BCA assay.

d. Wash streptavidin magnetic beads twice with RIPA lysis buffer and incubate them with the same volume of lysate from TurboID-*Cp*OGA^CD^ or control samples (TurboID-*Cp*OGA^DM^) on a rotator overnight at 4 °C.

e. Wash the beads twice with 1 mL of RIPA lysis buffer, once with 1 mL of 1 M KCl, once with 1 mL of 0.1 M Na_2_CO_3_, once with 1 mL of 2 M urea in 10 mM Tris-HCl (pH = 8.0), and twice with 1 mL RIPA lysis buffer.

f. Add protein loading buffer to the beads and heat at 95 °C for 10 min to elute the bound proteins for downstream analysis.

**C. Mass spectrometry analysis**

1. Separate the immunoprecipitated proteins using SDS-PAGE and then stain with Coomassie Brilliant Blue. Stop electrophoresis after proteins enter the separating gel for approximately 1 cm.

2. Cut the gel into small pieces and perform protein reduction using 10 mM DTT, followed by alkylation with 55 mM IAA.

3. Add 0.5 μg of sequencing-grade modified trypsin to the gel pieces and incubate overnight at 37 °C to digest the proteins.

4. Collect the digested peptides and desalt them using a StageTip, then dissolve the desalted peptides in 0.1% formic acid before proceeding to mass spectrometry.

5. Perform mass spectrometry analysis using the Q Exactive HF-X mass spectrometer and the Easy nLC 1200 system.

a. Use water (containing 0.1% formic acid) as mobile phase A and 80% acetonitrile (containing 0.1% formic acid) as mobile phase B.

b. Load the protein digestate directly onto the analytical column (75 μm × 15 cm, 1.9 μm C18, 1 μm tip) at a flow rate of 450 nL/min.

c. Collect data in data-dependent acquisition (DDA) mode, selecting the top 25 most intense precursor ions for MS/MS analysis.

## Data analysis

**Figure 6.**
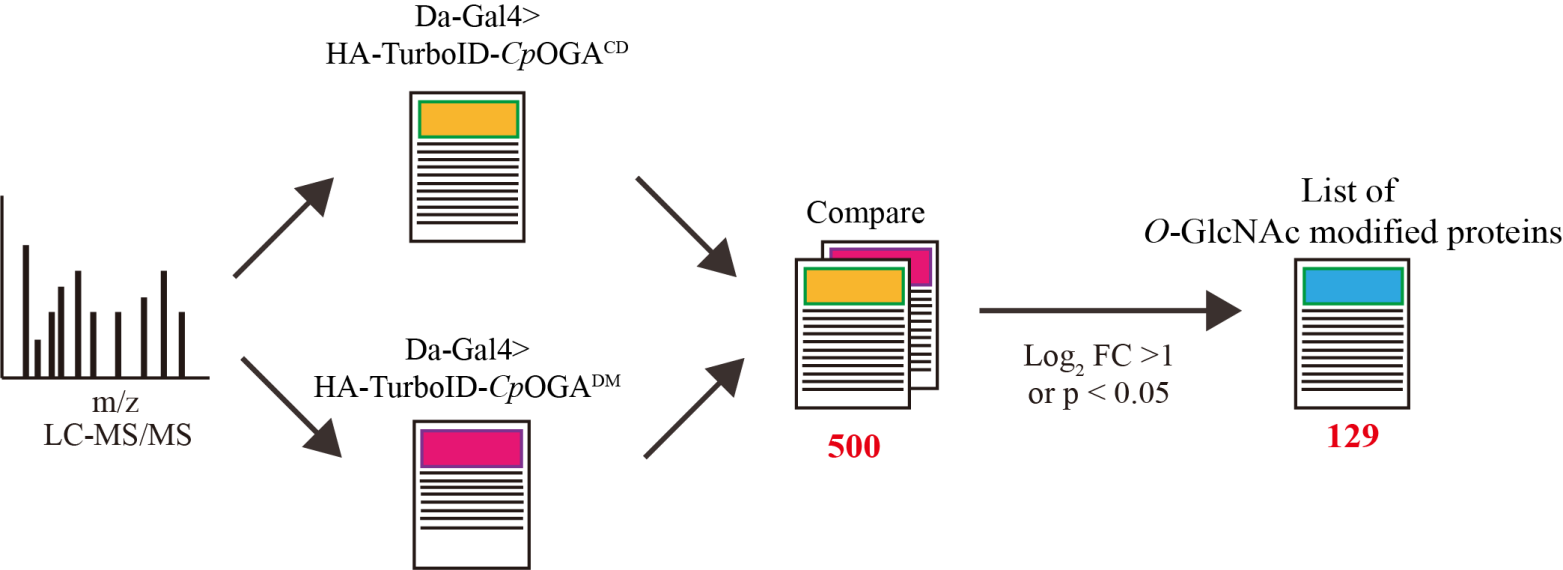
Overview of data processing. By comparing the mass spectrometry results, proteins that were selectively enriched (log_2_ FC > 1.5 or P <0.05) in the experimental group (TurboID-*Cp*OGA^CD^ group) relative to the control group (TurboID-*Cp*OGA^DM^ group) were regarded as O-GlcNAcylated substrates.

1. Import raw data into MaxQuant software for protein identification and quantification. Set the parameters as follows: 1) enzyme digestion by trypsin; 2) variable modifications include methionine oxidation, N-terminal acetylation, lysine and protein N-terminal biotinylation, and HexNAc (ST); 3) fixed modification as carbamidomethylation (C).

2. Use the *Drosophila* protein database to perform database searches. The database used should be the *Drosophila melanogaster* protein database, which includes both reviewed and unreviewed protein isoforms (22,088 total).

3. Set the false discovery rate (FDR) at both peptide-spectrum match (PSM) and protein levels to 1%. This FDR threshold ensures high confidence in the identification of peptides and proteins in the dataset.

4. Conduct label-free quantification (LFQ) of proteomic data from different *Drosophila* brain regions to determine the relative abundance of proteins across three replicates. The LFQ approach is used to assess protein levels based on ion intensities, allowing for a comparison of protein expression between different brain regions.

5. Use the Perseus software to filter out contaminants identified by MaxQuant. To avoid taking the logarithm of zero, add a pseudocount of 1 to protein intensities.

6. Calculate the log_2_ fold change (log_2_ FC) for each protein in the TurboID-*Cp*OGA^CD ^group relative to the TurboID-*Cp*OGA^DM ^control group.

7. For data from different *Drosophila* brain regions, select proteins identified in at least two of the three replicates in the TurboID-*Cp*OGA^CD^ group, and containing at least two peptides, for further analysis.

8. Assess the statistical significance of differences using a two-tailed unpaired Student’s t-test; proteins are identified as *O-*GlcNAcylated substrates if the log_2_ FC difference between the TurboID-*Cp*OGA^CD^ and TurboID-*Cp*OGA^DM^ groups is greater than 1 or if the difference is significant (p < 0.05) (Figure 6).

## General notes and troubleshooting

1. It is important to determine in advance the amount of protein required for mass spectrometry and the number of *Drosophila* needed to extract this amount.

2. The concentration of biotin and feeding duration used in steps A3b and A3c are the recommended conditions and can be adjusted based on the specific experiment to determine the optimal conditions.

## Validation of protocol

Yu et al. [15]. Tissue-specific O-GlcNAcylation profiling identifies substrates in translational machinery in Drosophila mushroom body contributing to olfactory learning. *eLife* 2024, 13: e91269. DOI:10.7554/eLife.91269.
